# Effects of SGLT2 inhibitors on cardiovascular outcomes in patients with stage 3/4 CKD: A meta-analysis

**DOI:** 10.1371/journal.pone.0261986

**Published:** 2022-01-12

**Authors:** Ning Li, Guowei Zhou, Yawei Zheng, Dan Lv, Xiangjun Zhu, Ping Wei, Min Zheng, Shijia Liu, Enchao Zhou, Wei Sun, Lu Zhang

**Affiliations:** Affilliated Hospital of Nanjing University of Chinese Medicine, Jiangsu Province Hospital of Chinese Medicine, Nanjing, China P.R; Scuola Superiore Sant’Anna, ITALY

## Abstract

**Introduction:**

After stage 3 CKD, the risk of adverse cardiovascular events increased significantly. Therefore, we performed a meta-analysis to investigate the cardiovascular protective effect of SGLT2 inhibitors in patients with stage 3/4 CKD with different baseline kidney function or underlying diseases.

**Method:**

To identify eligible trials, we systematically searched the Embase, PubMed, Web of Science, and Cochrane library databases from inception to April 15, 2021. The primary cardiovascular outcome was defined as a combination of cardiovascular mortality and hospitalization due to heart failure. Baseline kidney functions (stage 3a CKD: eGFR45-59mL/min per 1.73m2, stage 3b CKD: eGFR30-44mL/min per 1.73m2, stage 4 CKD: eGFR<30mL/min per 1.73m2) and underlying diseases (Type 2 diabetes, heart failure (Preserved ejection fraction or reduced ejection fraction), atherosclerotic cardiovascular disease) were used to stratify efficacy and safety outcomes. The results were subjected to a sensitivity analysis to ensure that they were reliable.

**Results:**

In the present study, a total of eleven trials were included that involved a total of 27,823 patients with stage 3/4 CKD. The treatment and control groups contained 14,451 and 13,372 patients, respectively. In individuals with stage 3/4 CKD, SGLT2 inhibitors reduced the risk of primary cardiovascular outcomes by 26% (HR 0.74, [95% CI 0.69–0.80], I^2^ = 0.00%), by 30% in patients with stage 3a CKD (HR 0.70, [95% CI 0.59–0.84], I^2^ = 18.70%), by 23% in patients with stage 3b CKD (HR 0.77, [95% CI 0.66–0.90], I^2^ = 2.12%), and by 29% in patients with stage 4 CKD (HR 0.71, [95% CI 0.53–0.96], I^2^ = 0.00%). The risk of primary outcomes was reduced by 29% (HR 0.71, [95% CI 0.63–0.80], I^2^ = 0.00%) in patients with type 2 diabetes, by 28% (HR 0.72, [95% CI 0.56–0.93], I^2^ = 37.23%) in patients with heart failure with preserved ejection fraction, by 21% (HR 0.79, [95% CI 0.70–0.89], I^2^ = 0.00%) in patients with heart failure with reduced ejection fraction, and by 25% (HR 0.75, [95% CI 0.64–0.88], I^2^ = 0.00%) in patients with atherosclerotic cardiovascular disease.

**Conclusions:**

For stage 3/4 CKD, SGLT2 inhibitors significantly decreased the risk of primary cardiovascular outcomes, and these benefits were consistent throughout the spectrum of different kidney functions, even in stage 4 CKD. There was no evidence of increased adverse outcomes across different baseline clinical complications, such as type 2 diabetes, heart failure, or atherosclerotic cardiovascular disease.

## 1. Introduction

Chronic kidney disease (CKD), which has a slow progressive clinical course and results in irreversible deterioration in renal function, has emerged as a major global health concern [1]. Alongside the deterioration of renal function, the risk of cardiovascular adverse events continues to increase [2, 3]. Cardiovascular complications were also discovered to be the leading cause of death among CKD patients [4]. As a result, preventing the progression of cardiovascular complications in CKD patients has become a global priority. Over the last two decades, guideline-directed medical therapy for cardiovascular disease has concentrated on reducing traditional and non-traditional risk factors, with a particular emphasis on inhibition of the renin-angiotensin-aldosterone system [5]. Several novel drugs have recently been developed to decrease the risk of cardiovascular outcomes [6–8], with sodium-glucose-cotransporter 2 (SGLT2) inhibitors ushering in a new era in CKD patients with cardiovascular complications.

In patients with type 2 diabetes or heart failure, SGLT2 inhibitors, a new class of glucose-lowering drugs, have been shown to reduce the risk of cardio-renal adverse effects [9]. In recent years, a succession of large-scale studies indicated that SGLT2 inhibitors can also reduce the risk of cardiovascular outcomes in patients with CKD, particularly those with type 2 diabetes [10, 11]. However, only a few studies have investigated the cardioprotective effects of SGLT2 inhibitors in patients with poor kidney function. There is widespread agreement that the risk of cardiovascular adverse events increases dramatically after stage 3 CKD (defined as an estimated glomerular filtration rate (eGFR) of <60 mL/min per 1.73m^2^) onwards [12, 13]. If SGLT2 inhibitors can be demonstrated to benefit such patients, the risk of death and their medical burden will be significantly reduced. Previous meta-analyses [14, 15] demonstrated the preliminary cardiovascular benefits of SGLT2 inhibitors in patients with stage 3/4 CKD. However, the populations included in these meta-analyses were all diabetic, and these studies failed to investigate the cardiovascular benefits in patients with varying baseline levels. Consequently, we decided to conduct a systematic review to gather more credible information on the cardiovascular benefits of SGLT2 inhibitors in patients with stage 3/4 CKD.

## 2. Methods

### 2.1 Study registration

The guideline of the Preferred Reporting Items for Systematic Reviews and Meta-Analysis (PRISMA) was used to develop and guide this systematic review and meta-analysis [16]. Furthermore, this meta-analysis was registered with the INPLASY database (INPLASY202180022). No ethical approval or patient consent was required given that all analyses were conducted based on previously published studies.

### 2.2 Search strategy

Four authors (NL, DL, XJZ, and PW) searched for randomized controlled trials that investigated the effectiveness of SGLT2 inhibitors. From their inception to August 27th, 2021, the following electronic databases were searched: PubMed, Web of Science, ScienceDirect, Embase, and Clinical trials (http://www.clinicaltrials.gov).

The search was conducted using medical subject headings (MeSH) along with free text terms, as well as Boolean logical operators. The following terms were used in the search: ("Sodium-Glucose Transporter 2 Inhibitors" OR “sodium glucose transporter ii inhibitor” OR “Sodium–glucose cotransporter 2 inhibitors” OR “SGLT-2 Inhibitors” OR “Inhibitor, SGLT-2” OR “Gliflozins” OR “Canagliflozin” OR “Dapagliflozin” OR “Empagliflozin” OR “Luseogliflozin” OR “Ipragliflozin” OR “Tofogliflozin” OR “Sotagliflozin” OR “Remogliflozin” OR “Sergliflozin” OR “Ertugliflozin”) AND “Randomized controlled trial”. To avoid omitting any eligible studies, any terms related to "SGLT2i" were searched.

Meanwhile, to minimize loss or omission of suitable articles that met our inclusion criterion, we conducted several exhaustive searches of major international conference proceedings, the grey literature (the noncommercial bibliography of doctors’ and masters’, technical documents (including government reports)) and clinical trials that may be ongoing or not yet published. Additionally, the references in each study and meta-analysis of SGLT2 inhibitors were also examined to avoid some studies that may be ignored. The search strategies supplement contains information on the databases and search strategies. A check was required to ensure the integrity and veracity of the research. To manage and verify the information, all records from the initial search were imported simultaneously into NoteExpress v3.2.0.7535 by four independent authors (NL, DL, YWZ, GWZ). During this procedure, discrepancies were resolved through discussion or mediation by a third author (LZ).

### 2.3 Study selection and prespecified outcome

#### 2.3.1 Study selection

The study inclusion criteria included: 1) Patients >18 years old with stage 3/4 CKD (Defined as having an estimated glomerular filtration rate (eGFR) of <60 mL/min/1.73 m^2^). 2) Trials that compared SGLT2 inhibitors to a placebo, with no dosage limitations. 3) Trials were restricted to parallel-group multicenter randomized controlled trials and at least a 6-month median follow-up. There were no language or regional restrictions. Repetitive studies, case reports, animal experiments, cohort studies, and retrospective studies were excluded. 4) studies that reported on cardiovascular outcomes of interest.

#### 2.3.2 Prespecified outcomes

The study’s primary cardiovascular outcomes included cardiovascular death or hospitalization for heart failure.

Secondary cardiovascular outcomes included: 1) 3P-MACE (three-point major adverse cardiovascular events: cardiovascular death, myocardial infarction, and stroke); 2) Hospitalization for heart failure; and 3) Cardiovascular death.

Other outcomes included: 1) Death from any cause; and 2) Safety outcome: any serious adverse outcome. The safety outcome was stratified based on the stage of the CKD.

### 2.4 Data extraction and quality assessment

Three authors (NL, YWZ, and GWZ) extracted the following information from each study: Sample size, age, publication year, study and population features, outcomes of interest, and period of treatment. Any discrepancies were resolved in consultation with an expert in this field (LZ). We obtained data that were not available in the original text or appendices by searching relevant secondary analyses or contacting the authors.

To assess the risk of bias in each trial, the Cochrane quality assessment tool provided by Review manager version 5.4 was used [17]. The risk of bias was examined independently by two authors (NL, YWZ). The assessment items included random sequence generation, allocation concealment, blinding of participants and personnel, blinding of the assessment of the outcome, an assessment of incomplete outcomes, incomplete outcome data, selective reporting, and other biases. Each item was assigned a risk level of unknown, low, or high. An analysis of the total bias for included studies was also measured. Any discrepancies were resolved by a third author (LZ).

### 2.5 Data analysis

To evaluate the effect of each trial, we pooled the Hazard ratio (HR) with 95% confidence intervals (CIs). Weighted mean differences (WMD) were used to analyze continuous variables. We also used a random-effects model with application of the DerSimonian–Laird method. We assessed heterogeneity between studies using the I^2^ statistic. Mild, moderate, and high heterogeneity were indicated by values of 25% or less, 25–50%, and 75% and more, respectively [18]. When more than 10 studies were included, we conducted a publication bias analysis using the Egger test [19]. For the sensitivity analysis, we eliminated each piece of literature one by one for analysis. We performed subgroup analyses on primary outcomes to verify if there were any differences between different eGFR subgroups (stage 3a CKD defined as eGFR 45–60 mL/min/1.73 m^2^, stage 3b CKD defined as eGFR 30–44 mL/min/1.73 m^2^, and stage 4 CKD defined as eGFR <30 mL/min/1.73 m^2^) and whether benefits changed in patients with different underlying diseases (such as type 2 diabetes, heart failure with preserved ejection fraction (HFpEF), heart failure with reduced ejection fraction(HFrEF), and atherosclerotic cardiovascular disease(ASCVD)). STATA version 16.0 was used to analyze the data.

## 3. Results

### 3.1 Study selection and features

**Fig 1:**
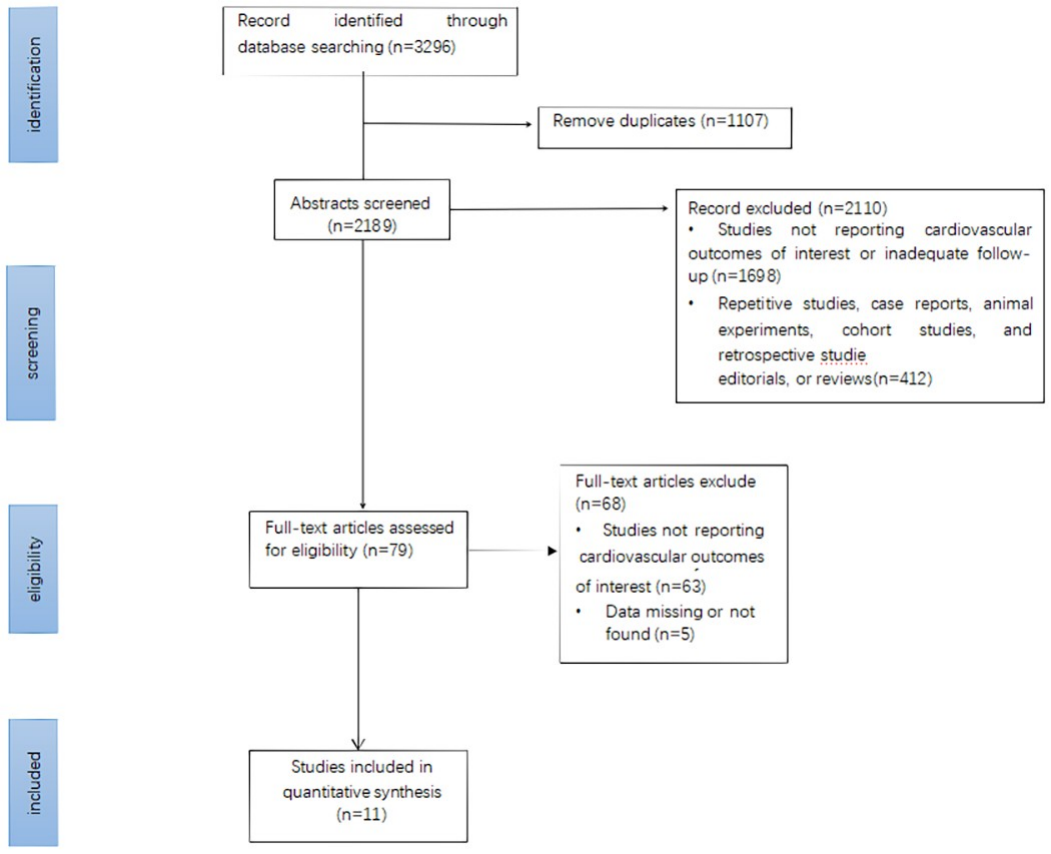
Identification of eligible studies: Flow diagram.

Searching the different databases yielded a total of 3,296 studies. After screening the abstracts and removing duplicates, 76 studies were retrieved. We performed full-text analyses of the studies, and a total of 11 trials were selected according to our strict selection criteria (Fig 1). Among them, four trials [20–23] involved patients with type 2 diabetes, two trials [11, 24] included patients with diabetic kidney disease, four trials [25–28] involved patients with heart failure, and three trials [10, 11, 24] included patients with chronic kidney disease. The attached file contains a detailed description of the screening and retrieval process. In all of the investigations, SGLT2 inhibitors were used as the intervention, with placebos being given to the control groups. All participants had stage 3/4 CKD. In total, 27,823 patients had eGFR <60 mL/min/1.73 m^2^, consisting of 14,451 and 13,372 people in the treatment and control group, respectively. The eGFR with the lowest value was 20 mL/min/1.73 m^2^. The average age of the patients in the trials varied from 61.8 to 70 years. The median follow-up time ranged from 9 to 42 months. Table 1 lists the characteristics of the studies that were included.

**Table 1:** Baseline characteristics among different studies.

Study	Study design	Setting	Drug dose (mg/day)	Median follow up (months)	eGFR (ml/min/1.73m^2^)	Age (yr)	Definition of cardiovascular outcomes
SGLT2i vs placebo							
CANVAS	RCT	Multinational	Canagliflozin 300/100	29	30–59	63.2±8.3/63.4±8.2	CV death or hospitalization for heart failure
CREDENCE	RCT	Multinational	Canagliflozin 100	31.4	30–59	62.9±9.2/63.2±9.2	CV death or hospitalization for heart failure
DAPA-CKD	RCT	Multinational	Dapagliflozin 10	28.8	25–45	61.8±12.1/61.9±12.1	CV death or hospitalization for heart failure
DAPA-HF	RCT	Multinational	Danagliflozin 10	18.2	30–59	66.2±11.0/66.5±10.8	CV death or HF hospitalization/urgent HF visit
DECLARE–TIMI 58	RCT	Multinational	Danagliflozin 10	50.4	<60	63.9±6.8/64.0±6.8	CV death or hospitalization for heart failure
EMPA-REG	RCT	Multinational	Empagliflozin 25/10	37.2	30–59	63.1 ± 8.6/63.2 ± 8.8	-
EMPEROR-Reduced	RCT	Multinational	Empagliflozin 10	16	20–59	67.2 ± 10.8/66.5 ± 11.2	CV death or hospitalization for heart failure
SCORED	RCT	Multinational	Sotagliflozin 200/400	16.0/15.9	25–59	69	CV death or HF hospitalization/urgent HF visit
SOLOIST-WHF	RCT	Multinational	Sotagliflozin 200/ 400	9.2/8.9	30–59	69/70	CV death or HF hospitalization/urgent HF visit
VERTIS CV	RCT	Multinational	Ertugliflozin 15/5	42	30–59	64.4±8.1/64.4±8.0	CV death or hospitalization for heart failure
EMPEROR-Preserved	RCT	Multinational	Empagliflozin 10	26.2	20–59	71.8±9.3/71.9±9.6	CV death or hospitalization for heart failure

RCT: Randomized controlled trials; eGFR:Estimated glomerular filtration rate; CV: Cardiovascular; HF: Hospitalization for heart failure; SGLT2i:SGLT2 inhibitors.

### 3.2 Evaluation of the quality of the included studies

Sufficient random sequence generation was observed in ten trials, and was unspecified in one trial [24]. All trials had adequate blinding of participants and employees. Only six trials [10, 20–22, 26, 27] mentioned allocation concealment, while this was unclear in the remaining studies. In all the trials, relative completeness in the evaluation of outcomes was demonstrated. The completeness of the outcome data in one trial [11] was unclear. Other biases in all of the studies that were unclear. Details on overall and individual biases are shown in the S1(A) and S1(B) Fig.

### 3.3 Primary outcome

**Fig 2:**
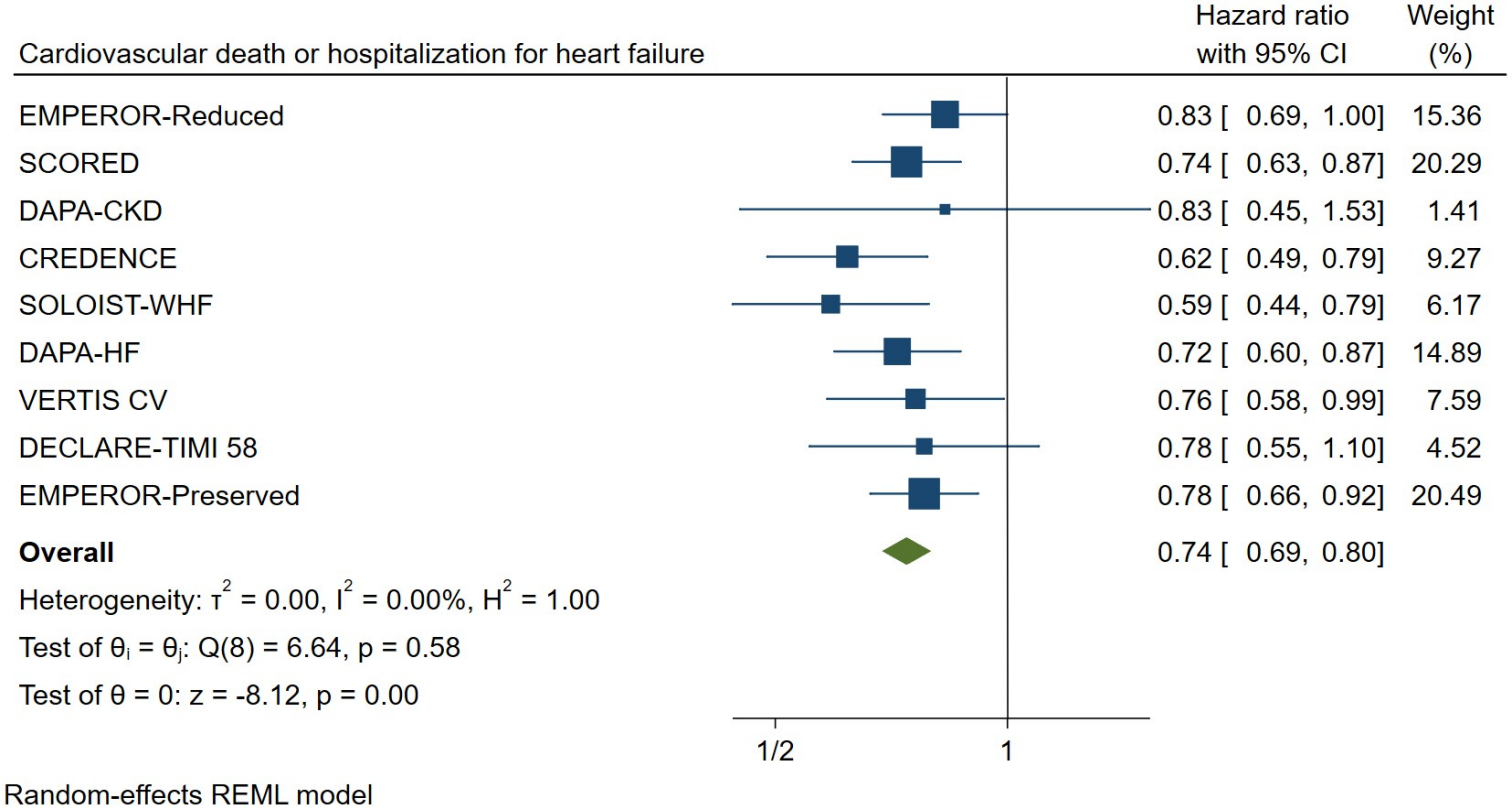
Effect of SGLT2 inhibitors on cardiovascular death or hospitalization for heart failure. CI: Confidence interval.

When compared to a placebo, SGLT2 inhibitors reduced the risk of primary cardiovascular outcomes by 26% (HR 0.74, [95% CI 0.69–0.80], I^2^ = 0.00%) in patients with stage 3/4 CKD (Fig 2).

#### 3.3.1 eGFR subgroups

**Fig 3:**
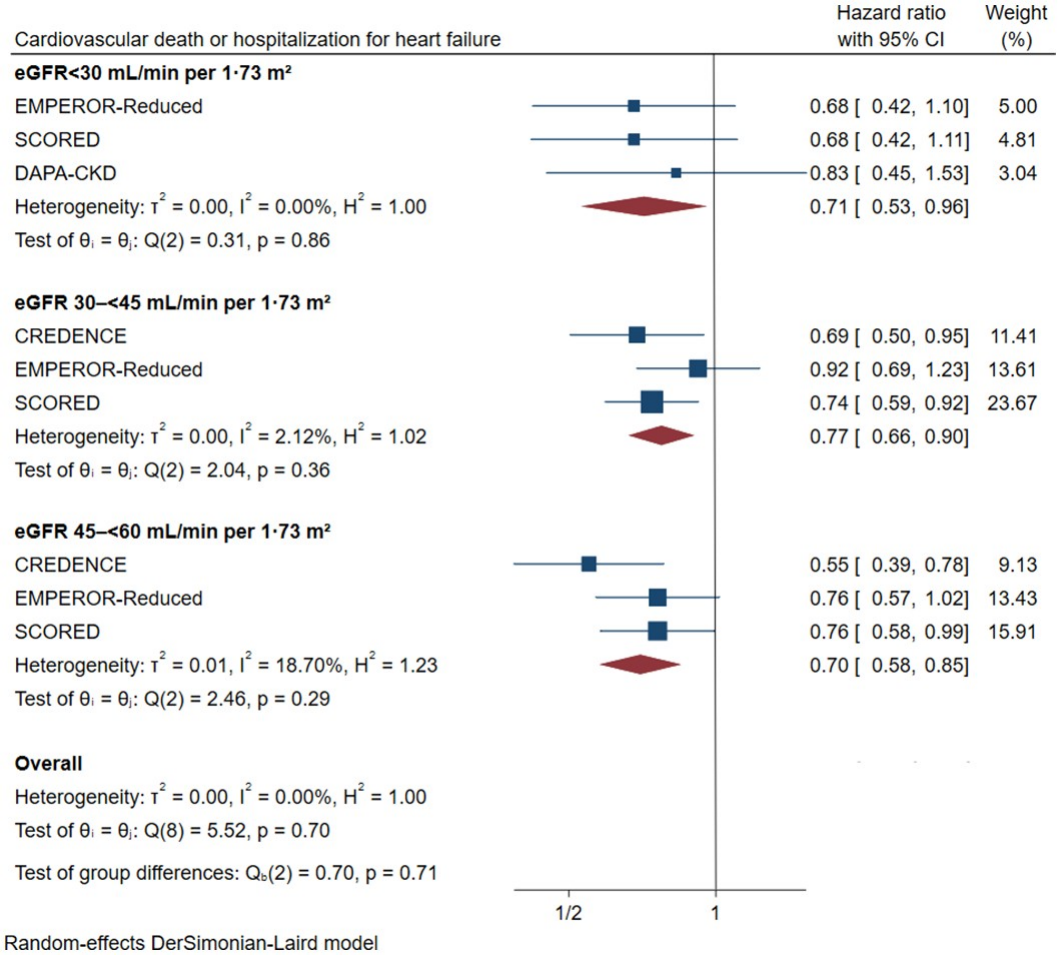
Effect of SGLT2 inhibitors on cardiovascular death or hospitalization for heart failure across the spectrum of different levels of eGFR. CI: Confidence interval; eGFR: estimated glomerular filtration rate.

In different subgroups of eGFR, SGLT2 inhibitors lowered the risk of the primary cardiovascular outcome (Fig 3). The HR was reduced by 30% in patients with stage 3a CKD, (HR 0.70, [95% CI 0.58–0.85], I^2^ = 18.70%), by 23% in patients with stage 3b CKD (HR 0.77, [95% CI 0.66–0.90], I^2^ = 2.12%) and by 29% in patients with stage 4 CKD (HR 0.71, [95% CI 0.53–0.96], I^2^ = 0.00%). The cardiovascular benefit was consistent across different stages (P interaction = 0.71).

#### 3.3.2 Subgroups for different underlying diseases

**Fig 4:**
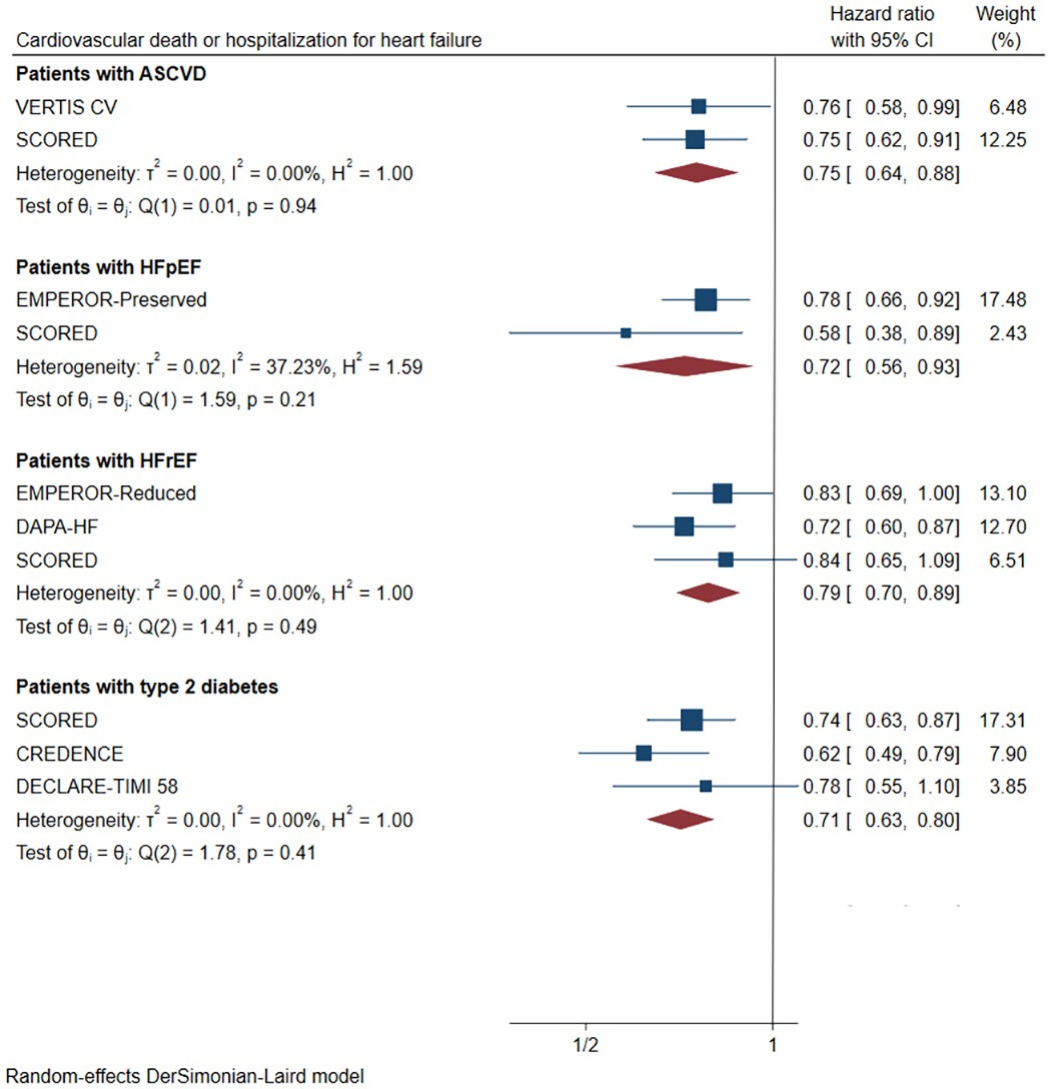
Effect of SGLT2 inhibitors on cardiovascular death or hospitalization for heart failure in patients with different underlying diseases. CI: confidence interval; HFpEF: preserved ejection fraction; HFrEF: reduced ejection fraction; ASCVD: atherosclerotic cardiovascular disease.

*3*.*3*.*2*.*1 Patients with type 2 diabetes*. For patients with type 2 diabetes and stage 3/4 CKD, SGLT2 inhibitors reduced the risk of the primary cardiovascular outcome by 29% (Fig 4, HR 0.71, [95% CI 0.63–0.80], I^2^ = 0.00%).

*3*.*3*.*2*.*2 Patients with heart failure (HFpEF)*. In patients with stage 3/4 CKD and with heart failure (HFpEF), SGLT2 inhibitors significantly decreased the risk of the primary cardiovascular outcome (Fig 4, HR 0.72, [95% CI 0.56–0.93], I^2^ = 37.23%).

*3*.*3*.*2*.*3 Patients with heart failure (HFrEF)*. In patients with stage 3/4 CKD combined with heart failure (HFrEF), SGLT2 inhibitors reduced the risk of the primary cardiovascular outcome by 21% (Fig 4, HR 0.79, [95% CI 0.70–0.89], I^2^ = 0.00%).

*3*.*3*.*2*.*4 Patients with ASCVD*. For patients with ASCVD and stage 3/4 CKD, SGLT2 inhibitors reduced the risk of the primary cardiovascular outcome by 25% (Fig 4, HR 0.75, [95% CI 0.64–0.88], I^2^ = 0.00%).

### 3.4 Secondary outcomes

#### 3.4.1 Secondary cardiovascular outcomes

SGLT2 inhibitors reduced the risk of the 3P-MACE by 18% (S2 Fig) in patients with stage 3/4 CKD (HR 0.82, [95% CI 0.72–0.93], I^2^ = 37.47%). The HR of hospitalization due to heart failure was also significantly reduced (S2 Fig) by 34% (HR 0.66, [95% CI 0.59–0.74], I^2^ = 0.00%). The risk of cardiovascular death was reduced by 13% (S2 Fig, HR 0.87, [95% CI 0.77–0.98], I^2^ = 0.00%).

### 3.5 Other outcomes

**Fig 5:**
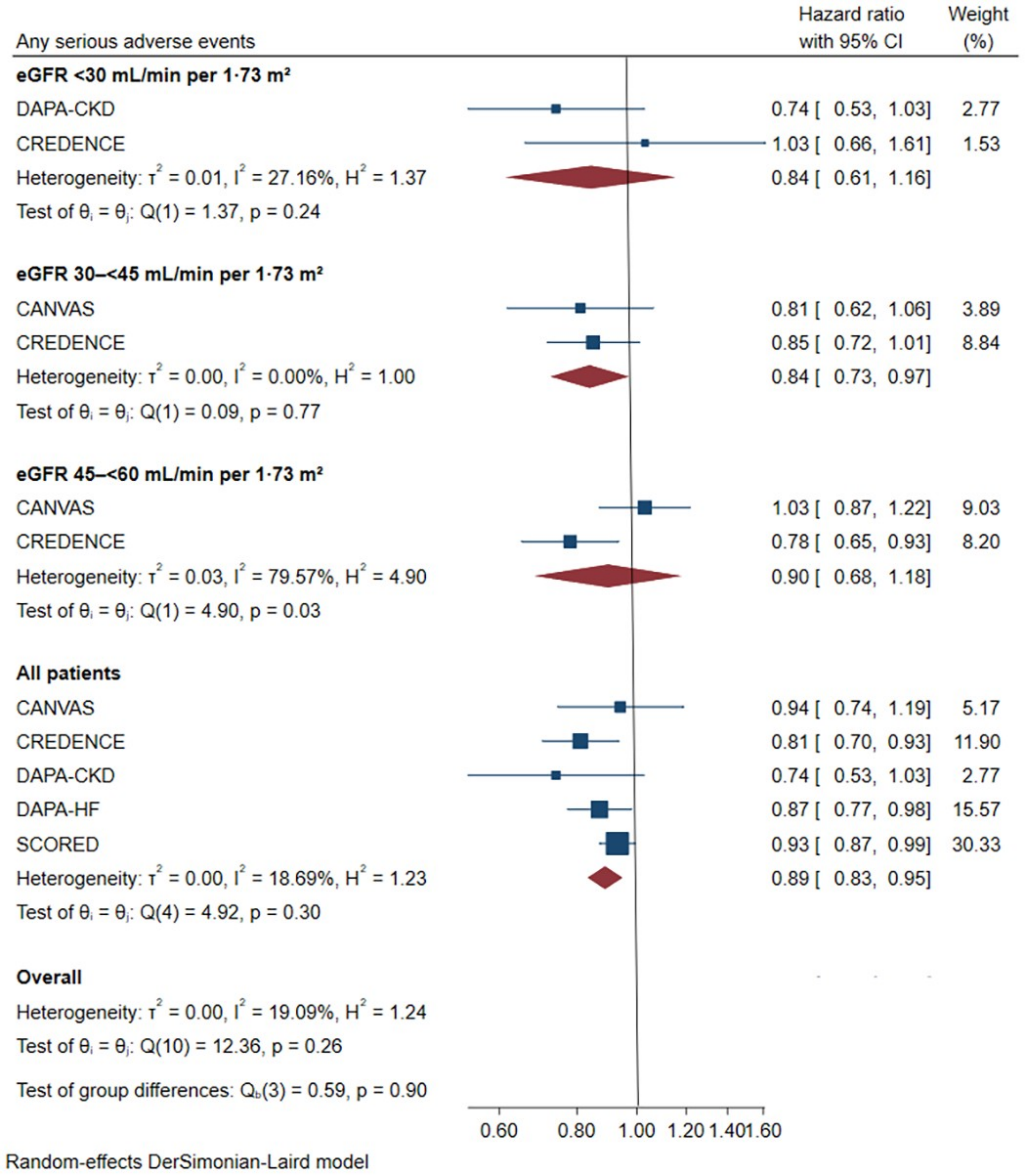
Effect of SGLT2 inhibitors on serious adverse outcome. CI: confidence interval; eGFR: estimated glomerular filtration rate.

According to our results, there were no significant differences in the reduction of all-cause death when compared to a placebo (S2 Fig, HR 0.92, [95% CI 0.81–1.04], I^2^ = 0.00%). In terms of safety, the risk of the serious adverse outcome was reduced by 11% (Fig 5, HR 0.89, [95% CI 0.83–0.95], I^2^ = 18.69%). The risk of adverse event did not increase with the stage of the CKD (stage 3a: HR 0.90, [95% CI 0.68–1.18], I^2^ = 79.57%; stage 3b: HR 0.84, [95% CI 0.73–0.97], I^2^ = 0.00%; stage4: HR 0.84, [95% CI 0.61–1.16], I^2^ = 27.16%).

### 3.6 Sensitivity analysis

After excluding the literature one by one, the confidence interval for the primary outcome fluctuated between 0.67 and 0.81, which confirmed the robustness of the result of primary cardiac outcome (S3 Fig).

## 4. Discussion

Our meta-analysis provides evidence, based on current clinical trials; for the effectiveness and safety of SGLT2 inhibitors on cardiovascular outcomes in patients with stage 3/4 CKD with different baseline kidney function or underlying diseases. Our results indicated that SGLT2 inhibitors could significantly reduce the risk of hospitalization due to heart failure or cardiovascular death in patients with stage 3/4 CKD (HR 0.74, [95% CI 0.69–0.80]), which further confirms the cardiovascular benefit of SGLT2 inhibitors in patients with poor kidney function. Additionally, the risk of 3P-MACE, hospitalization for heart failure, and cardiovascular death were all significantly reduced, which demonstrated that SGLT2 inhibitors have wide application prospects in preventing the occurrence and development of cardiac complications in patients with poor kidney function.

Since SGLT2 inhibitors antagonize glucose reabsorption in renal tubules, their activity is likely to be eGFR-dependent [29]. The effectiveness and safety of SGLT2 inhibitors in patients with low eGFR are unknown. Previously, 277 patients with stage 4 CKD were included in the study. The results demonstrated that the sotagliflozin group had fewer adverse cardiovascular events than in the placebo group [30]. Our study indicated that patients with stage 3a or 3b CKD had a much lower risk of adverse cardiovascular events. These benefits were also seen in patients with stage 4 CKD. A post-hoc analysis study [31] on canagliflozin demonstrated that the cardiovascular protection effect was consistent in individuals with stage 4 CKD and eGFR ≥30 mL/min/1.73 m^2^. A predetermined analysis [32] of dapagliflozin yielded similar findings. The two studies mentioned above indicated that patients with stage 4 CKD may benefit from continued application of SGLT2 inhibitors. Despite this, the findings of these two studies were not statistically significant. In contrast, the current study suggests that using SGLT2 inhibitors in stage 4 CKD not only lowered the risk of cardiovascular outcomes, but also did not raise the risk of serious adverse events. As a result, we recommend that patients with stage 4 CKD continue to take SGLT2 inhibitors until they begin maintenance dialysis or receive a kidney transplant. Some mechanisms independent of eGFR may be responsible for the CV benefits of SGLT2 inhibitors. A new study [33] has found that the inhibitory effect of SGLT2 inhibitors on the kidney and cardiac sodium hydrogen exchanger 3 (NHE3) may be associated with a corresponding reduction in cardiac injury, systolic dysfunction, and hypertension. It can play a certain role in cardiovascular protection. In addition, some anti-inflammatory mechanisms of SGLT2 inhibitors may also play a role in cardiovascular protection [34]. More cardiovascular protective mechanisms that are not dependent on eGFR need to be investigated. The current EMPA-Kidney study (ClinicalTrials.gov: NCT03594110) involves patients with eGFR ranging from 20–45 ml/min per 1.73 m^2^. We believe that the results of this trial may provide additional insight into cardio-kidney benefits in patients with low eGFR.

For patients with different underlying diseases, our meta-analysis revealed that the risk of primary cardiovascular outcome was considerably reduced regardless of whether they had type 2 diabetes or cardiovascular disease. The SCORED trial [28] included patients with stage 3/4 CKD and type 2 diabetes, it demonstrated that sotagliflozin reduced the risk of hospitalization due to heart failure or cardiovascular death. Similar results could be confirmed when integrated with other large scale trials in our meta-analysis. The DAPA-CKD prespecified analysis demonstrated that dapagliflozin lowered the risk of cardio-renal outcome in CKD patients independent of the presence of cardiovascular illness [35]. The subgroup analysis of the CREDENCE trial also revealed that the treatment with canagliflozin in patients with type 2 diabetes and CKD, with or without cardiovascular disease, appeared to provide a consistent cardio-renal benefit [36]. To further investigate the cardiovascular protective effect in patients with CKD and poor kidney function, we divided the group with cardiovascular disease into those with heart failure (cardiorenal syndrome (CRS)) and those with atherosclerotic cardiovascular disease. For patients with CRS, the risk of hospitalization for heart failure or cardiovascular death was significantly reduced whether ejection fraction was preserved or reduced. These results imply that the cardiovascular benefits of CRS patients with varying levels of ejection fraction remained constant. CRS is a bidirectional disorder in which the heart and kidney may induce or perpetuate disease in the other organ [37, 38], and the risk of death is substantially higher than in a single disease [39]. The GWTG-HF trial [40] included 365,494 patients with heart failure and discovered that when eGFR was below 60mL/min per 1.73m^2^, the incidence of adverse cardiovascular events was significantly increased. In individuals with CRS [41], cardiac resynchronization therapy (CRT), β-blockers, RAASi, and angiotensin receptor neprilysin inhibitor (ARNI) are all recommended as major treatments. The results of our study suggest that SGLT2 inhibitors could be a new option in the treatment of CRS, particularly in patients with an eGFR <60 mL/min/1.73 m^2^. For patients with ASCVD, a secondary analysis from the ENCORE trial demonstrated that decreased eGFR is associated with a greater risk of adverse ASCVD events [42]. The REACTION study [43] produced the same result. In patients with stage 3/4 CKD and ASCVD, our meta-analysis showed that SGLT2 inhibitors reduced the risk of adverse cardiovascular outcomes. However, because all of the enrolled patients with ASCVD had type 2 diabetes, further high-quality studies are needed to evaluate the efficacy of SGLT2 inhibitors in patients with ASCVD who do not have diabetes.

According to emerging evidence, the main mechanism of the cardio-protection effect is thought to be in promoting the excretion of urine sodium and sugar, which reduces the pre-after load of cardiac and improves myocardial remodeling [44]. In addition, the effect of enhanced myocardial energetics, increased red-cell mass, improved oxygen supply, weight loss, reductions in blood pressure, adaptive cellular reprogramming, and reduced glomerular perfusion pressure by regulating tubulo-glomerular feedback, delay the progression of kidney failure and may also lead to a cardiovascular benefit [45–48]. More research is needed to determine the exact mechanism.

Our meta-analysis had limitations. First, we used combined data rather than individual participant data. Second, there were discrepancies in endpoint definitions in some studies, which may have had an adverse impact on our results. Third, the patients included in the studies had different underlying diseases, and the majority of the data came from subgroup analyses of large trials. This may weaken the credibility of the results of the present study.

## 5. Conclusion

SGLT2 inhibitors significantly reduced the risk of primary cardiovascular outcomes in patients with stage 3/4 CKD, and this benefit was consistent across all stages, including stage 4. Furthermore, consistent significant cardiovascular benefits were reported in patients with type 2 diabetes, heart failure or ASCVD. For safety outcomes, SGLT2 inhibitors did not increase the number of serious adverse events, even in patients with stage 4 CKD. According to our results, SGLT2 inhibitors are the latest addition to the toolbox of therapies used to manage CKD patients with poor kidney function. We believe that SGLT2 inhibitors may be used safely in patients with stage 3/4 CKD, even if their eGFR <30 mL/min per 1.73m^2^.

## Supporting information

S1 Fig Quality evaluation of the included literature. (A) Risk of bias in the included studies. The authors reviewed the risk of bias for each item in each included study. (B) Risks of bias of individual studies. +: low risk of bias; −: high risk of bias; ?: unclear risk of bias.
(PNG)

S2 Fig Effect of SGLT2 inhibitors on secondary outcome. CI: confidence interval; Worsening kidney function: defined as doubling of serum creatinine or sustained 40% decline in eGFR; kidney failure: defined as requirement for chronic dialysis or kidney transplantation, or sustained eGFR <15 mL/min/1.73 m^2^. eGFR: estimated glomerular filtration rate.
(PNG)

S3 Fig Sensitivity outcomes. (PNG)

S1 Dataset (XLS)

S1 Checklist (DOCX)
