# Supplementary figures and images for: Effects of SGLT2 inhibitors on cardiovascular outcomes in patients with stage 3/4 CKD: A meta-analysis
Source: PLoS One. 2022 Jan 12;17(1):e0261986. doi: 10.1371/journal.pone.0261986 (PMC8754287; doi:10.1371/journal.pone.0261986)

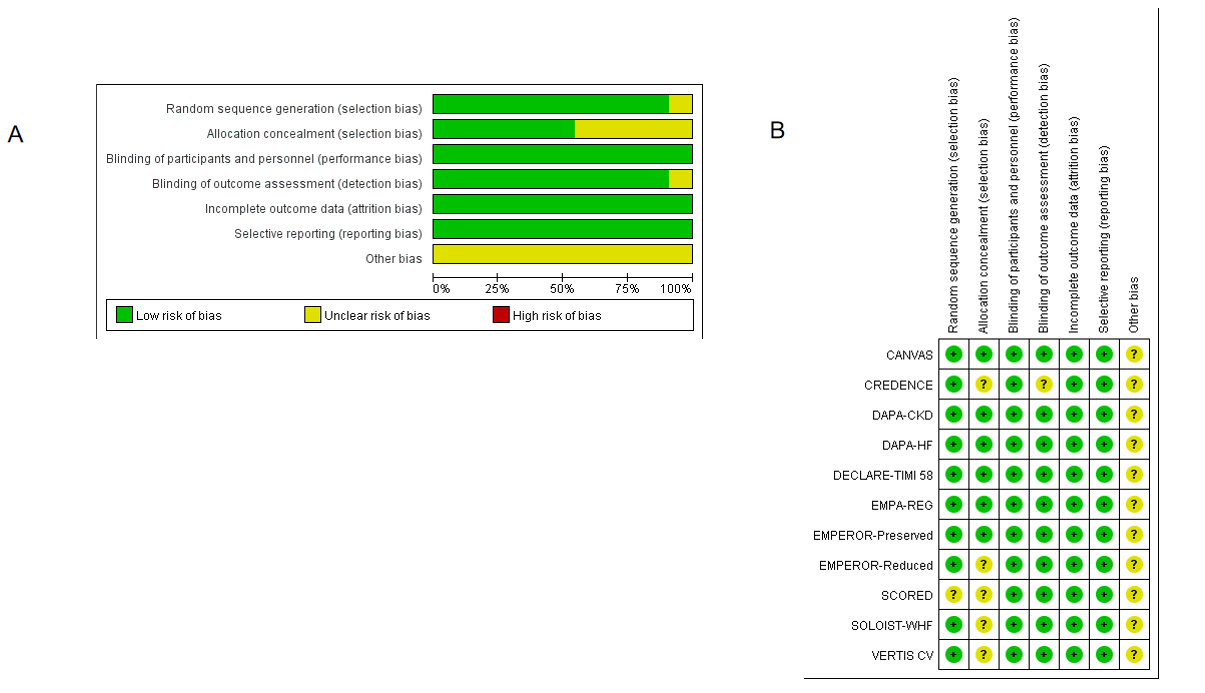

Supplement: S1 Fig — (A) Risk of bias in the included studies. The authors reviewed the risk of bias for each item in each included study. (B) Risks of bias of individual studies. +: low risk of bias; −: high risk of bias; ?: unclear risk of bias. (PNG) [file pone.0261986.s001.png]

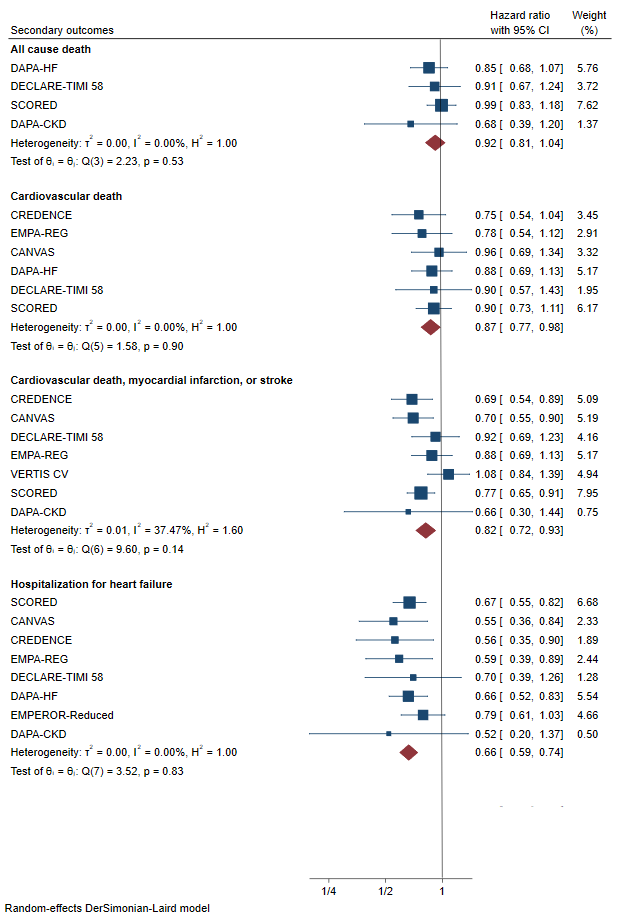

Supplement: S2 Fig — CI: confidence interval; Worsening kidney function: defined as doubling of serum creatinine or sustained 40% decline in eGFR; kidney failure: defined as requirement for chronic dialysis or kidney transplantation, or sustained eGFR <15 mL/min/1.73 m2. eGFR: estimated glomerular filtration rate. (PNG) [file pone.0261986.s002.png]

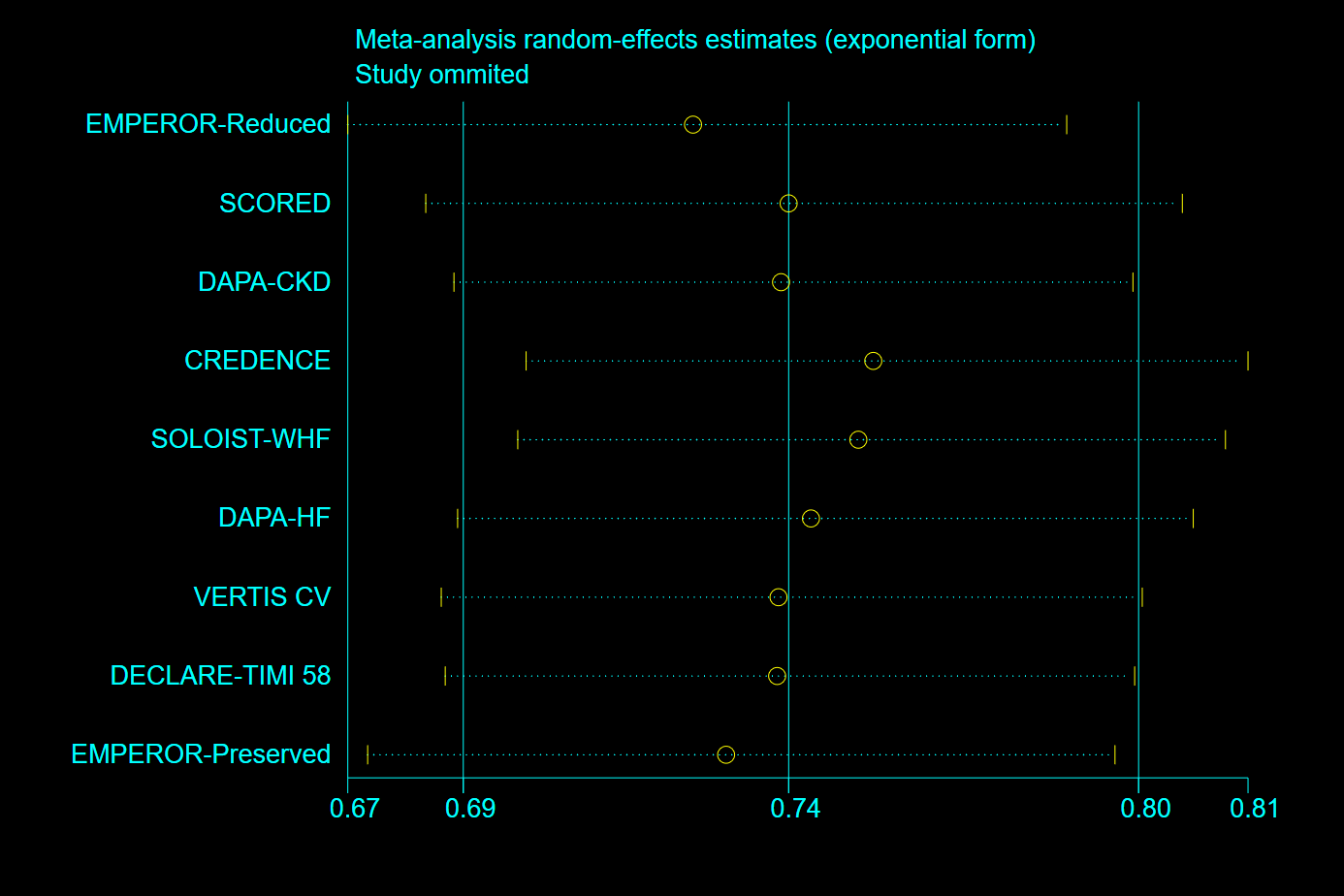

Supplement: S3 Fig — (PNG) [file pone.0261986.s003.png]
